# Brain Functional Mechanisms Determining the Efficacy of Transcutaneous Auricular Vagus Nerve Stimulation in Primary Insomnia

**DOI:** 10.3389/fnins.2021.609640

**Published:** 2021-03-12

**Authors:** Xiao Wu, Yue Zhang, Wen-ting Luo, Run-ru Mai, Xiao-yan Hou, Zi-qiang Xia, Bi-yun Xu, Bo Liu

**Affiliations:** ^1^Department of Chinese Medicine, Sichuan Provincial People’s Hospital, University of Electronic Science and Technology of China, Chengdu, China; ^2^Department of Radiology, The Second Affiliated Hospital of Guangzhou University of Chinese Medicine, Guangzhou, China; ^3^The Second Clinical College of Guangzhou University of Chinese Medicine, Guangzhou, China; ^4^Department of Sleep Disorder, Fangcun Branch, The Second Affiliated Hospital of Guangzhou University of Chinese Medicine, Guangzhou, China

**Keywords:** primary insomnia, transcutaneous auricular vagus nerve stimulation (taVNS), fractional amplitude of low-frequency fluctuations (fALFF), heart rate variability (HRV), bio-markers, efficacy

## Abstract

Transcutaneous auricular vagus nerve stimulation (taVNS) has been reported to be effective in the treatment of primary insomnia (PI); however, its efficacy varies considerably across individuals for reasons that are unclear. In order to clarify the underlying mechanisms, this study investigated the effects of taVNS on spontaneous neuronal activity and autonomic nervous system function by functional magnetic resonance imaging (fMRI) and measurement of heart rate variability (HRV), respectively, in patients with PI. Forty patients with PI were divided into effective (group A) and ineffective (group B) groups based on their response to taVNS as determined by Pittsburgh Sleep Quality Index score reduction rate (group A ≥ 25% and group B < 25%). Spontaneous neuronal activity was measured by fractional amplitude of low-frequency fluctuations (fALFF) and HRV values and was compared between the two groups as well as before *vs* after taVNS. We then analyzed the correlations among efficacy of taVNS for 4 weeks, the fALFF and HRV values during continuous taVNS state. The results showed that the HRV parameter values (i.e., root mean square of successive differences, percentage of adjacent NN intervals differing by >50 ms, and high frequency) of group A were higher than those of group B during continuous taVNS state. In the fMRI scan, the fALFF values of the right cerebellum, right medial superior frontal gyrus, and bilateral supplementary motor area—which belong to the sensorimotor network (SMN)—were lower in group A than in group B during continuous taVNS state. The correlation analysis revealed that the efficacy of continuous taVNS and HRV and fALFF values were interrelated. These findings demonstrate that differential regulation of the SMN by the autonomic nervous system may be responsible for inter-individual variations in the efficacy of taVNS and suggest that HRV and fALFF are potential biomarkers for predicting PI patients’ response to taVNS treatment.

## Introduction

Primary insomnia (PI) is a common chronic disease that is mainly treated by cognitive behavioral therapy for insomnia (CBT-I) and sedative–hypnotic drugs (Patel et al., 2018). However, CBT-I has shortcomings, including limited efficacy in preventing sleep attacks, inconvenience, long treatment duration, and high cost (Kay-Stacey and Attarian, 2016), whereas drugs have adverse effects such as delirium, endocrine disorder, anterograde memory disorder, and daytime sleepiness (Kay-Stacey and Attarian, 2016). As such, there is an urgent need for new therapies for insomnia that have minimal side effects and can be used over a long term.

Transcutaneous auricular vagus nerve stimulation (taVNS) is known to be effective in the treatment of insomnia (Luo et al., 2017; Jiao et al., 2020; Zhao et al., 2020). Patients with PI often exhibit autonomic nervous system imbalance (Javaheri and Redline, 2017), elevated levels of neurogenic inflammatory factors (Irwin et al., 2016), and hypersensitivity to external noise or emotional stimulation (Lauriola et al., 2019). taVNS directly attenuates autonomic nerve imbalance (Laborde et al., 2019), activates the cholinergic neuron system, and inhibits the release of inflammatory mediators (De Couck et al., 2014). Imaging studies have revealed that taVNS modulates neuronal activity in the periaqueductal gray, hypothalamus, thalamus, and hippocampus, which are brain areas that are closely related to sleep disorders (Badran et al., 2018a).

The efficacy of taVNS for the treatment of insomnia varies considerably across individuals (Li et al., 2019; Song et al., 2019). In one study, taVNS was effective in 80% of PI patients (Li et al., 2019), whereas in hemodialysis patients with insomnia, the rate was 69.49% (Song et al., 2019). Although taVNS has been approved by the US Food and Drug Administration (FDA) for the treatment of severe epilepsy and major depression (Johnson and Wilson, 2018; Kong et al., 2018), the efficacy rates in these diseases are also highly variable (Englot et al., 2016; Song et al., 2018). Clarifying the reasons for the differences in efficacy is important for developing personalized treatments that can improve clinical success rates and reduce medical costs.

Heart rate variability (HRV) is an objective and frequently used parameter to gauge the activity of the autonomic nervous system, including the autonomic response to taVNS (Chapleau and Sabharwal, 2011; Sacha, 2014; Williams et al., 2019). The vagus nerve shows variable sensitivity to VNS (Hagen et al., 2014), which is reflected by differences in the degree of alteration in HRV in response to the same stimulation parameters (Badran et al., 2018b). Patients showing greater changes in HRV during VNS have a better clinical outcome (Englot et al., 2016). Moreover, children with sleep disorders or anxiety often have lower vagus nerve tension than their normal peers, with tension of the vagus nerve shown to be negatively correlated with clinical symptoms (El-Sheikh et al., 2007).

Amplitude of low-frequency fluctuations (ALFF) or fractional (f)ALFF is an important parameter in functional magnetic resonance imaging (fMRI) that reflects the activity in a given brain region and has been used to explore the neural basis of insomnia and monitor treatment response (Zou et al., 2008; Liu et al., 2016; Zhao et al., 2020). We speculated that the variable efficacy of continuous taVNS among patients with PI is due to differences in autonomic responses and brain activity. In this study, we used fALFF and HRV as metrics for neural activity and autonomic activation, respectively, in order to clarify the mechanisms underlying inter-individual differences in the efficacy of taVNS for the treatment of insomnia. We compared fALFF and HRV values before and during continuous taVNS and analyzed the correlations between taVNS efficacy and fALFF and HRV values in patients with PI.

## Materials and Methods

### Patients

This study was registered as a part of the “Research for the brain regulation mechanisms of acupoint stimulation in the region of auricular vagus nerve for insomnia patients based on fMRI clinical trial” (clinical trial number: ChiCTR1900022535). A total of 77 patients with PI were recruited from the Department of Sleep Disorder, Fangcun Branch, The Second Affiliated Hospital of Guangzhou University of Chinese Medicine (Guangdong Provincial Hospital of Chinese Medicine) through advertisements to outpatients. The study was approved by the Ethics Committee of Guangdong Provincial Hospital of Chinese Medicine. All the patients signed an informed consent form before voluntarily participating in the study.

Primary insomnia was diagnosed according to the Fifth Edition of the American Diagnostic and Statistical Manual of Mental Disorders (Taylor et al., 2018) criteria by two experienced psychiatrists from the Department of Sleep Disorder. Any discrepancies in diagnosis were resolved through discussion or with input from more senior neurosurgeons. The patients met the following inclusion criteria (Huls et al., 2018; Zhao et al., 2020): (1) age 18–65 years and right-handed, (2) at least one of the three typical symptoms of insomnia (i.e., difficulty in falling asleep, difficulty in maintaining a sleep state, or insufficient sleep time) lasting > 3 months and meeting the diagnostic criteria of PI, (3) Pittsburgh Sleep Quality Index (PSQI) score > 7, Self-Rating Anxiety Scale (SAS) score < 59, and Self-Rating Depression Scale (SDS) score < 62, (4) patients with no nervous system disease, metal implants in the body, claustrophobia, or any other contraindication for MRI, and (5) education level of at least junior high school (in order to be able to understand the scales). The exclusion criteria (Huls et al., 2018) were as follows: (1) patients with a history of head injury, nervous system diseases, drug abuse, and other objective environmental factors that could cause insomnia symptoms, (2) patients with tumors or other serious primary diseases of the hematopoietic or endocrine system, (3) PSQI score ≤ 7, SAS score ≥ 59, or SDS score ≥ 62, (4) patients with contraindications for vagus nerve stimulation such as arrhythmia and asthma, (5) allergic reaction in or damage to the skin of the stimulation area, and (6) participation in other clinical trials in the previous 6 months or taking anticholinergic or other drugs that could inhibit cerebral cortex or nervous system activity at the time of the examination.

### Clinical Evaluation and Grouping

According to the “Guiding Principles for Clinical Research of New Drugs of Chinese Medicine” that was published by the National Health Commission of China, treatment efficacy was determined by the PSQI score reduction rate after taVNS treatment, which was calculated with the following formula:

$$\mathrm{Score}\,\mathrm{reduction}\,\mathrm{rate}=\frac{P\,S\,Q\,I\,\left(b\,e\,f\,o\,r\,e\,\_\,t\,r\,e\,a\,t\,m\,e\,n\,t\right)-P\,S\,Q\,I\,\left(a\,f\,t\,e\,r\,\_\,t\,r\,e\,a\,t\,m\,e\,n\,t\right)}{P\,S\,Q\,I\,\left(b\,e\,f\,o\,r\,e\,\text{\_}\,t\,r\,e\,a\,t\,m\,e\,n\,t\right)}\times 100\%.$$

Patients with a PSQI score reduction rate ≥ 25% and <25% were assigned to the effective group (group A) and ineffective group (group B), respectively (Song et al., 2016; Kwon et al., 2018).

### Treatment

**FIGURE 1:**
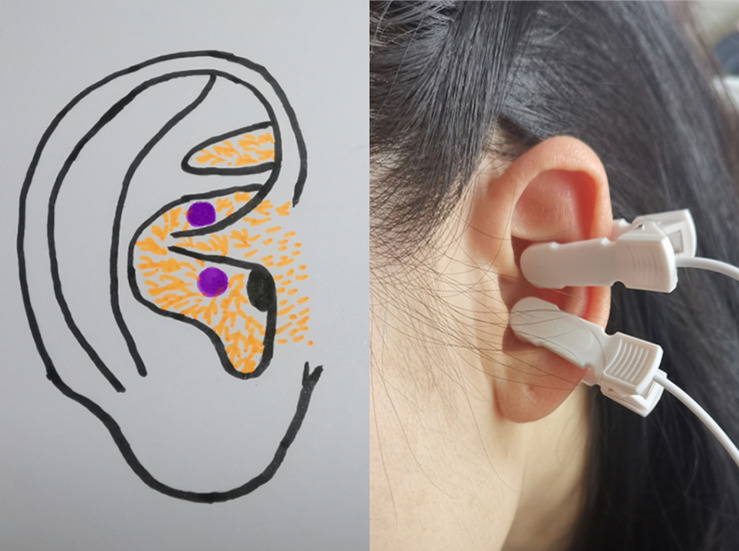
Site of transcutaneous auricular vagus nerve stimulation stimulation on the auricular surface.

A taVNS device (SDZ-IIB; Hwato, Suzhou, China) was used to stimulate the auricular cavum concha of the vagus nerve (Figure 1) with the following parameters (Rong et al., 2016; Kong et al., 2018; Zhao et al., 2020): (1) density waves adjusted to 20 Hz, with a wave width of 0.2 ms, (2) intensity adjusted based on the tolerance of the patient (7–12 mA), and (3) similar to a previous study design, the taVNS treatments consisted of 40 sessions (two sessions a day, 5 days per week for 4 weeks).

### MRI Data Acquisition

fMRI scanning was performed on a 3.0-T Ingenia MR scanner (Philips, Amsterdam, Netherlands) with a 32-channel birdcage head coil. The subject was told to remain awake and motionless and avoid having any thoughts, with the eyes closed during the scan. Each scanning session lasted approximately 20 min. The scan included a high-resolution anatomical image, an 8-min resting state (rs-)fMRI scan before taVNS, and an 8-min fMRI scan during continuous taVNS state.

Functional images were obtained with the following parameters: field of view (FOV) = 240 × 240 × 142 mm, voxel size = 3.75 × 3.75 × 3.5 mm, matrix = 64 × 61 × 38 slices, repetition time (TR) = 2,000 ms, echo time (TE) = 30 ms, dynamic scans = 240, slices = 38, slice gap = 0.25 mm; patient position = head first, patient orientation = supine, frequency-encoding direction = anterior–posterior, flip angle = 90, speeder phase encoding = 1, fastest pulse = off, slice orientation = sagittal, and scan time = 8:06 s.

The parameters for three-dimensional high-resolution full-brain structural images (T1) were as follows: FOV = 256 × 240 × 224 mm, voxel size = 0.8 × 0.8 × 0.8 mm, matrix = 320 × 300 × 280 slices, TR = 1.0 ms, TE = 4.7 ms, dynamic scans = 240, slices = 280, slice gap = 0 mm, patient position = head first, fastest pulse = off, patient orientation = supine, frequency-encoding direction = anterior–posterior, flip angle = 8°, slice orientation = sagittal, scan time = 5:14 s, and peripheral nerve stimulation mode = moderate.

### HRV Data Acquisition

Heart rate variability was measured using the cardiac electrical gating device on the MR scanner. A script compiled by the instrument manufacturer was used to calculate the HRV value before and during continuous taVNS state.

### Statistical Analysis

#### rs-fMRI Data Processing

The resting-state fMRI data preprocessing was performed using DPABI 13.0 (Yan et al., 2016) based on MATLAB (The Math Works, Natick, MA, United States). For each subject, the data were processed by the following seven steps: (1) among the 240 volumes, the first 10 functional volumes were discarded for magnetization equilibrium, and the other 230 volumes were used for further analysis; (2) slice-timing correction was conducted to adjust the acquisition time delay between slices; (3) the functional images were realigned to the first volume for correction of head movement, and there were no subjects with excessive head motion (>2.5 mm of displacement or >2.5° of rotation in any direction); (4) the functional images were co-registered to the individual sMRI and spatially normalized to standard MNI space using DARTEL; (5) the normalized functional images were resampled to 3 mm × 3 mm × 3 mm and smoothed with a Gaussian kernel at a full width at half maximum of 6 mm; (6) a regression model was applied to regress out the nuisance variables (head motion parameters from the Friston 24-parameter model, white matter signal, cerebro-spinal fluid signal) from the time series of each voxel, and the linear trends were removed; (7) fALFF calculation: the time series of each voxel was transformed into a frequency domain, and the power spectrum was obtained using the fast Fourier transform. The square root was measured at each frequency of the power spectrum, and the averaged square root, i.e., ALFF value, was acquired over the range of 0.01–0.10 Hz. The fALFF value was obtained by dividing the total ALFF values from 0.01 to 0.025 Hz (Zou et al., 2008).

#### Statistical Analysis of fMRI Data

The intergroup analysis (group A *vs.* group B in continuous stimulation state) of the fALFF was performed using a two-sample *t*-test, with the mean head-motion value (mean FD Jenkinson) as covariate variables. A threshold of voxel-wise *p* uncorrected and a cluster-level *p* corrected by family wise error were applied for multiple-comparison corrections. If voxel-wise *P* < 0.005 and cluster-level *P* < 0.05, the difference was statistically significant. Besides this, in the baseline resting state and continuous stimulation state, we respectively extracted the average *z* values of significantly altered clusters (group A *vs.* group B in continuous stimulation state). Then, the differences of the fALFF values were compared using a two-sample *t*-test between group A and group B in the baseline resting state and continuous stimulation state, respectively, and *P* < 0.05 was considered to be statistically significant.

We also explored the association among the altered fALFF values (baseline minus continuous stimulation state), HRV (continuous stimulation state), and the efficacy assessments (difference in PSQI score before *vs.* after 1 month of taVNS) across all subjects after Bonferroni correction.

#### Statistical Analysis of Clinical Indicators

Statistical analyses were performed with SPSS v18.0 for Windows (IBM Corp, Armonk, NY, United States), and differences with *P* < 0.05 were considered as statistically significant. Continuous or numerical variables are expressed as mean ± SD. The Shapiro–Wilk and Levene tests were used to test the normality and homogeneity of variances, respectively. Normally distributed variables with assumed homogeneity of variance were compared with the independent-samples *t*-test, and non-normally distributed variables or those without homogeneous variance were compared with the Mann–Whitney *U* test. Categorical variables are expressed as the composition ratio or rate, and comparisons were performed with Pearson’s chi-square or Fisher’s exact test. Pearson and Spearman sequential correlation analyses were carried out for normally and non-normally distributed variables, respectively. Data conforming to normal and non-normal distributions are presented as a histogram and a box plot, respectively.

## Results

### Demographic Characteristics and PSQI Scores

**FIGURE 2:**
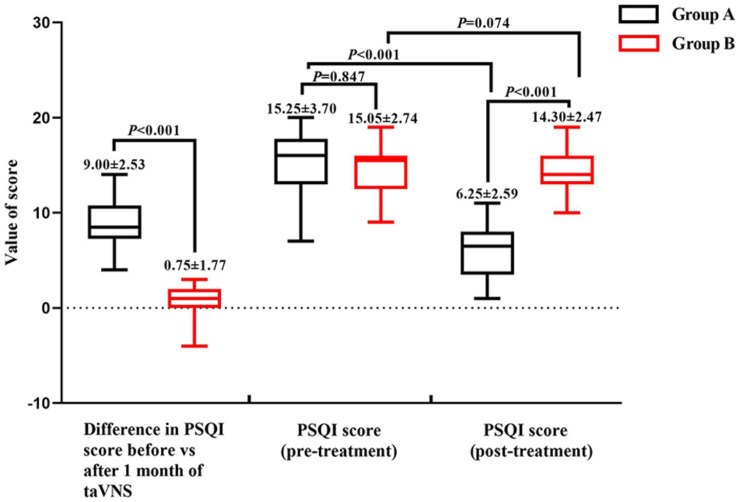
Pittsburgh Sleep Quality Index scores of groups A and B before and after treatment.

The demographic characteristics and PSQI scores of the study population are shown in Table 1. There were no significant differences in demographic data between the two groups. The mean PSQI score of group A was significantly lower than that of group B, and the difference in PSQI score before *vs.* after treatment was larger for group A than for group B, indicating that taVNS treatment was more effective in the former (Figure 2).

**TABLE 1:** Demographic and baseline clinical characteristics of the study population.

Item	Group A (*n* = 20)	Group B (*n* = 20)	*t*/*Z*/*χ*^2^	*P*
Age, years	49.40 ± 12.22	46.20 ± 12.76	–0.810	0.379*
Weight, kg	58.28 ± 9.30	56.78 ± 8.27	0.537	0.594*
Height, cm	161.35 ± 9.56	162.75 ± 6.70	0.536	0.595*
BMI	22.33 ± 3.07	21.35 ± 2.12	1.170	0.250*
Blood pressure, mmHg				
SBP	123.40 ± 15.95	123.5 ± 11.90	–0.022	0.982*
DBP	74.10 ± 11.77	77.4 ± 6.75	–1.088	0.285*
Insomnia duration, months (range)	37.00 (17.25–112.50)	87.00 (15.00–120.00)	–0.939	0.348^&^
Sex, *n* (%)				
Male	5 (25.0)	7 (35.0)	0.476	0.490^#^
Female	15 (75.0)	13 (65.0)		
Smoking, *n* (%)				
Yes	0 (0)	0 (0)	–	1.000^△^
No	20 (100)	20 (100)		
Drinking, *n* (%)				
Yes	2 (10)	1 (5)	0.000	1.000^□^
No	18 (90)	19 (95)		
Hypertension, *n* (%)				
Yes	1 (5)	1 (5)	0.000	1.000^□^
No	19 (95)	19 (95)		
Diabetes, *n* (%)				
Yes	0 (0)	0 (0)	–	1.000^△^
No	20 (100)	20 (100)		
Allergy, *n* (%)				
Yes	5 (25.0)	2 (10.0)	0.693	0.405^□^
No	15 (75.0)	18 (90.0)		
Hyperlipemia, *n* (%)				
Yes	0 (0)	0 (0)	–	1.000^△^
No	20 (100)	20 (100)		

Data are shown as mean ± SD, unless otherwise indicated. *Independent-samples *t*-test. ^&^Mann–Whitney *U* test. ^#^Pearson’s chi-square test. ^□^Pearson’s chi-square test (continuity correction). ^△^Fisher’s exact probability test. BMI, body mass index; DBP, diastolic blood pressure; SD, standard deviation; SBP, systolic blood pressure.

### HRV Results

**FIGURE 3:**
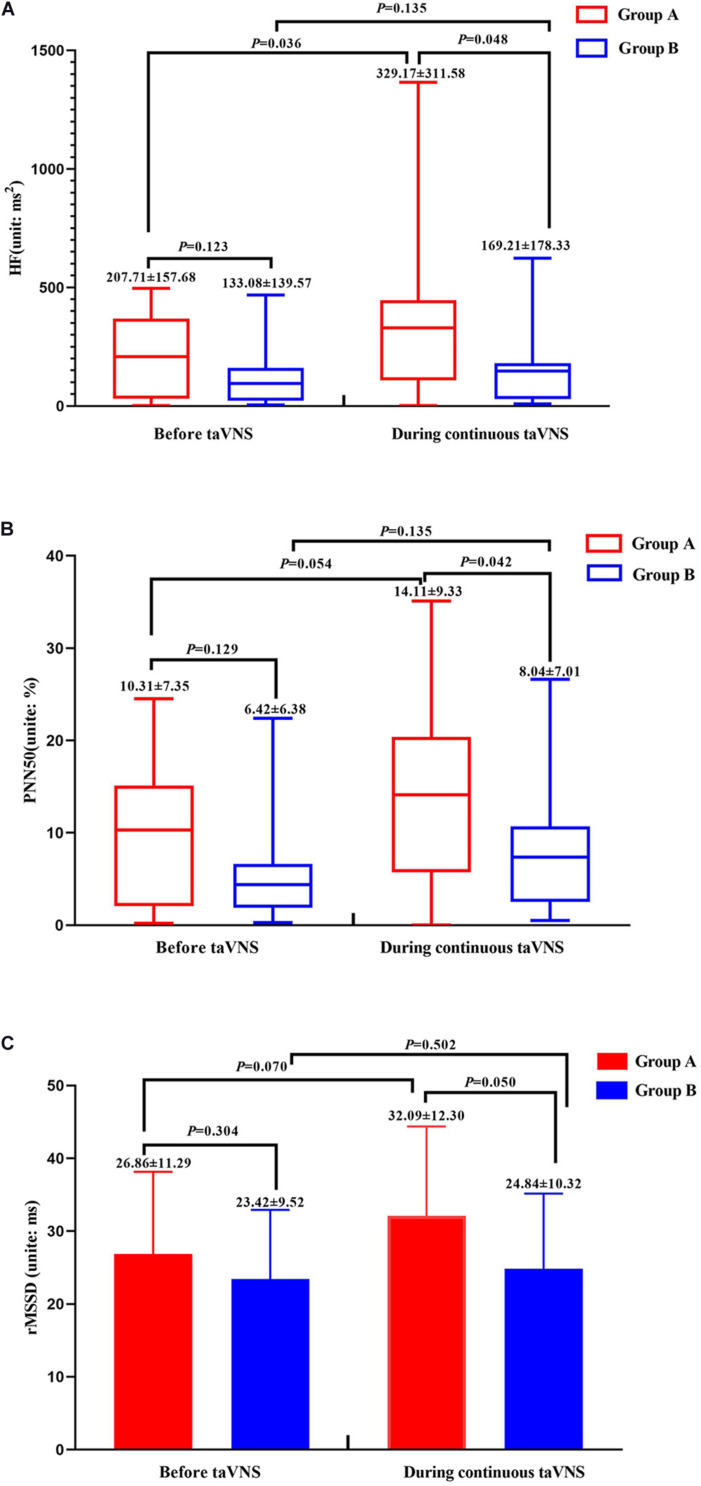
Heart rate variability parameters of groups A and B before and during continuous transcutaneous auricular vagus nerve stimulation state. HF **(A)**, PNN50 **(B)**, and rMSSD **(C)** of groups A and B.

The values of the HRV parameters HF and PNN50 were higher for group A than for group B (*P* < 0.05) during continuous taVNS state; although there was no significant difference between the two groups in terms of the value of rMSSD, there was a trend of a higher value for group A (*P* = 0.05). There were no significant differences in these three parameters before taVNS (Figure 3).

### fMRI Results

**FIGURE 4:**
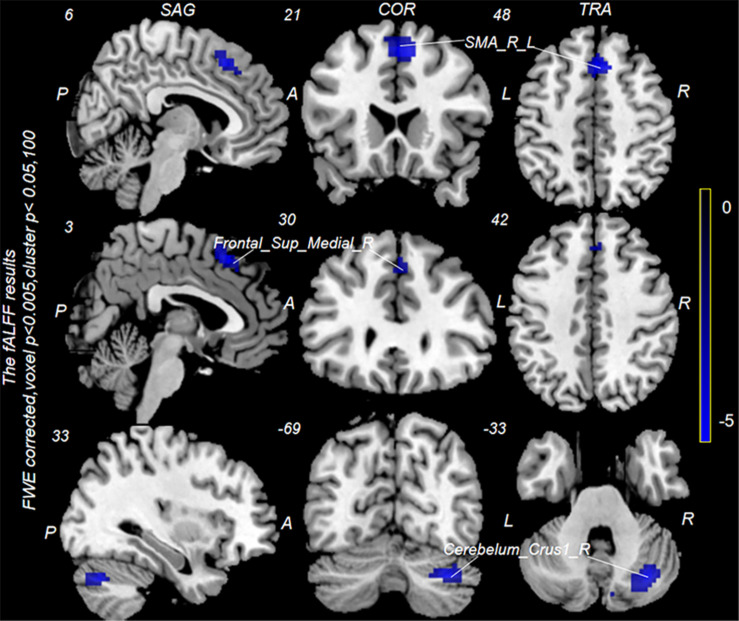
Changes in fractional amplitude of low-frequency fluctuation (fALFF) values in groups A and B during continuous transcutaneous auricular vagus nerve stimulation (taVNS) state. Cool colors indicate regions with decreased fALFF values during continuous taVNS state.

**FIGURE 5:**
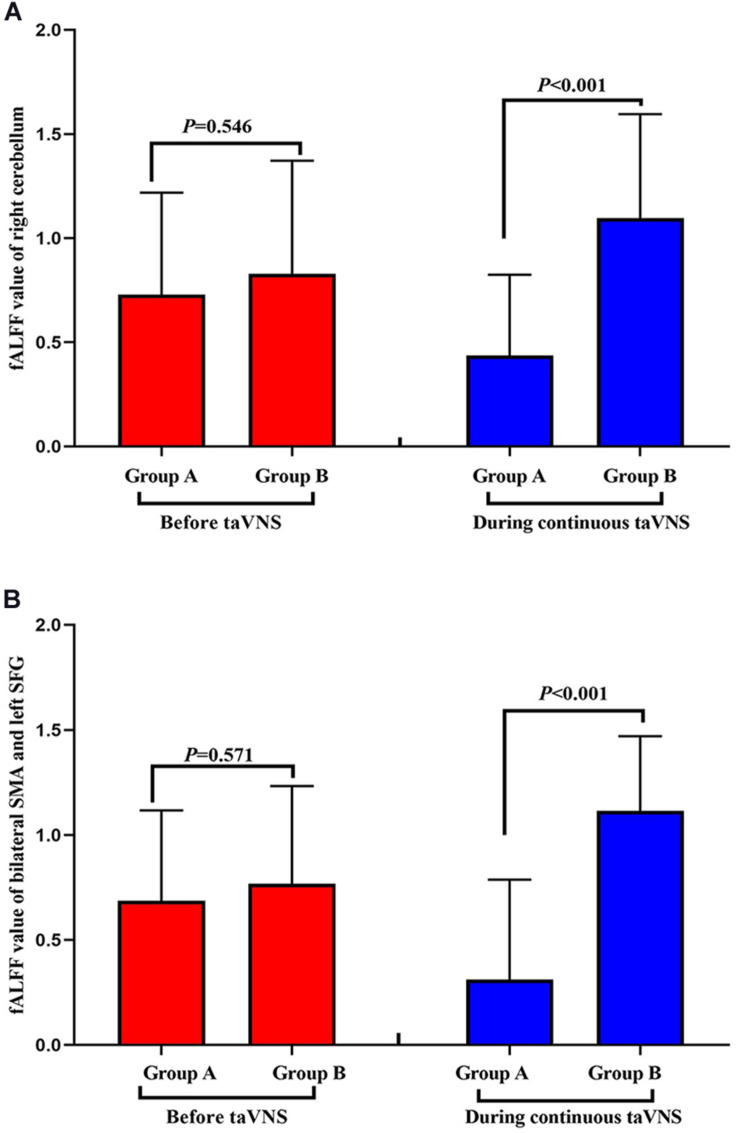
Comparison of the fractional amplitude of low-frequency fluctuation (fALFF) values of groups A and B before and during transcutaneous auricular vagus nerve stimulation (taVNS). fALFF value of right cerebellum **(A)** and bilateral SMA and left SFG **(B)**.

Neuronal activity as reflected by the fALFF value was significantly decreased in the right cerebellum, right medial superior frontal gyrus (mSFG), and bilateral supplementary motor area (SMA) during continuous taVNS state in group A (Figure 4 and Table 2). These brain regions showed no significant differences in fALFF value before taVNS (Figure 5). Thus, brain activity in the sensorimotor network (SMN) of group A was significantly lower than that of group B (*P* < 0.005) during continuous taVNS state.

**TABLE 2:** Brain regions differing significantly between groups A and B during continuous transcutaneous auricular vagus nerve stimulation treatment.

Contrast	Cluster	Brain region	Peak *T* value	Peak *Z* value	MNI coordinates		
					*X*	*Y*	*Z*
Effective > ineffective	–	No brain region above the threshold					
Effective < ineffective	115	Right supplementary motor area	4.90	4.26	6	21	48
		Left supplementary motor area	3.83	3.48	−6	12	63
		Right medial superior frontal gyrus	2.96	2.71	3	30	42
	100	Right superior cerebellum	4.84	4.22	33	−69	−33

*MNI, Montreal Neurological Institute.*

### Correlation Analyses

**FIGURE 6:**
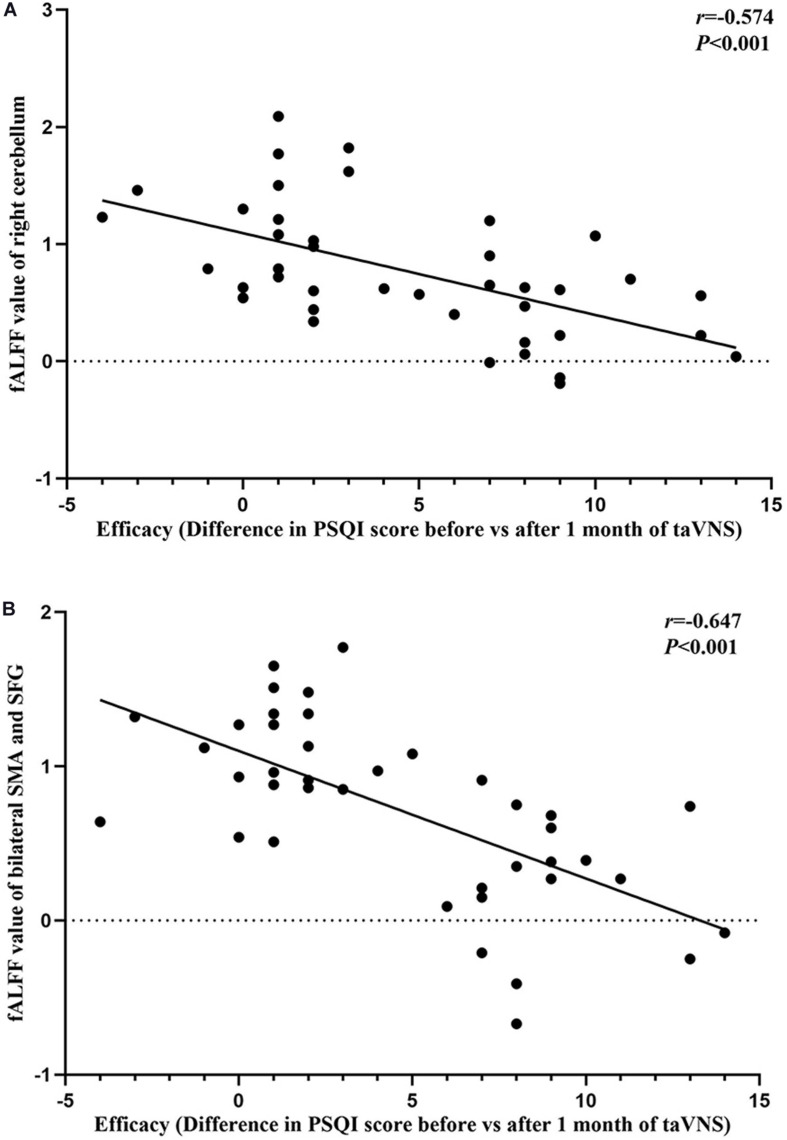
Results of the correlation analysis between transcutaneous auricular vagus nerve stimulation (taVNS) efficacy (difference in Pittsburgh Sleep Quality Index score before *vs.* after 1 month of taVNS) and fractional amplitude of low-frequency fluctuation (fALFF) values. **(A,B)** Correlations between taVNS efficacy and fALFF values of the right cerebellum **(A)** and the combination of bilateral supplementary motor area and superior frontal gyrus **(B)**.

**FIGURE 7:**
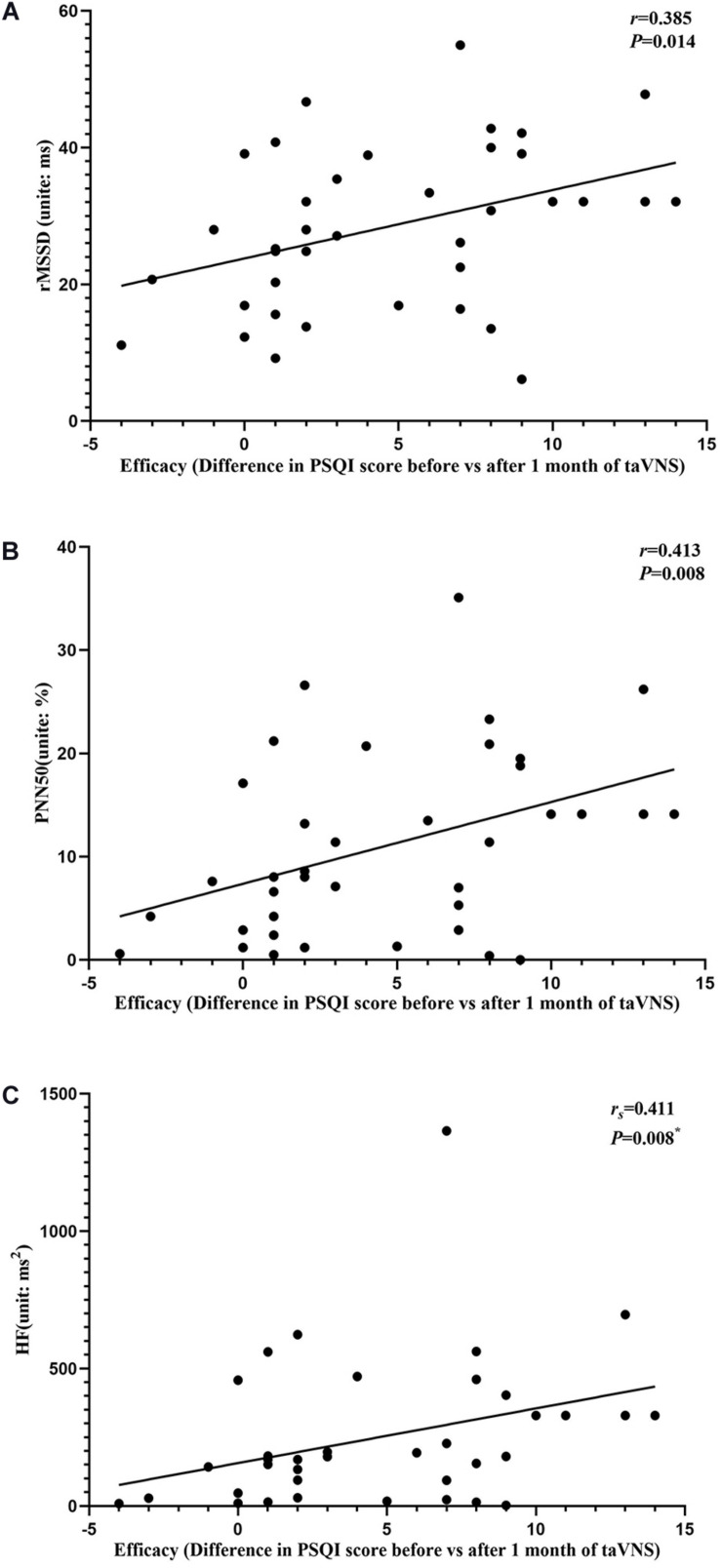
Results of the correlation analysis between transcutaneous auricular vagus nerve stimulation (taVNS) efficacy (difference in Pittsburgh Sleep Quality Index score before *vs.* after 1 month of taVNS) and heart rate variability parameters. **(A–C)** Correlations between efficacy and rMSSD **(A)**, PNN50 **(B)**, and HF **(C)**. The asterisk means *P*-value for Spearman’s correlation analysis.

**FIGURE 8:**
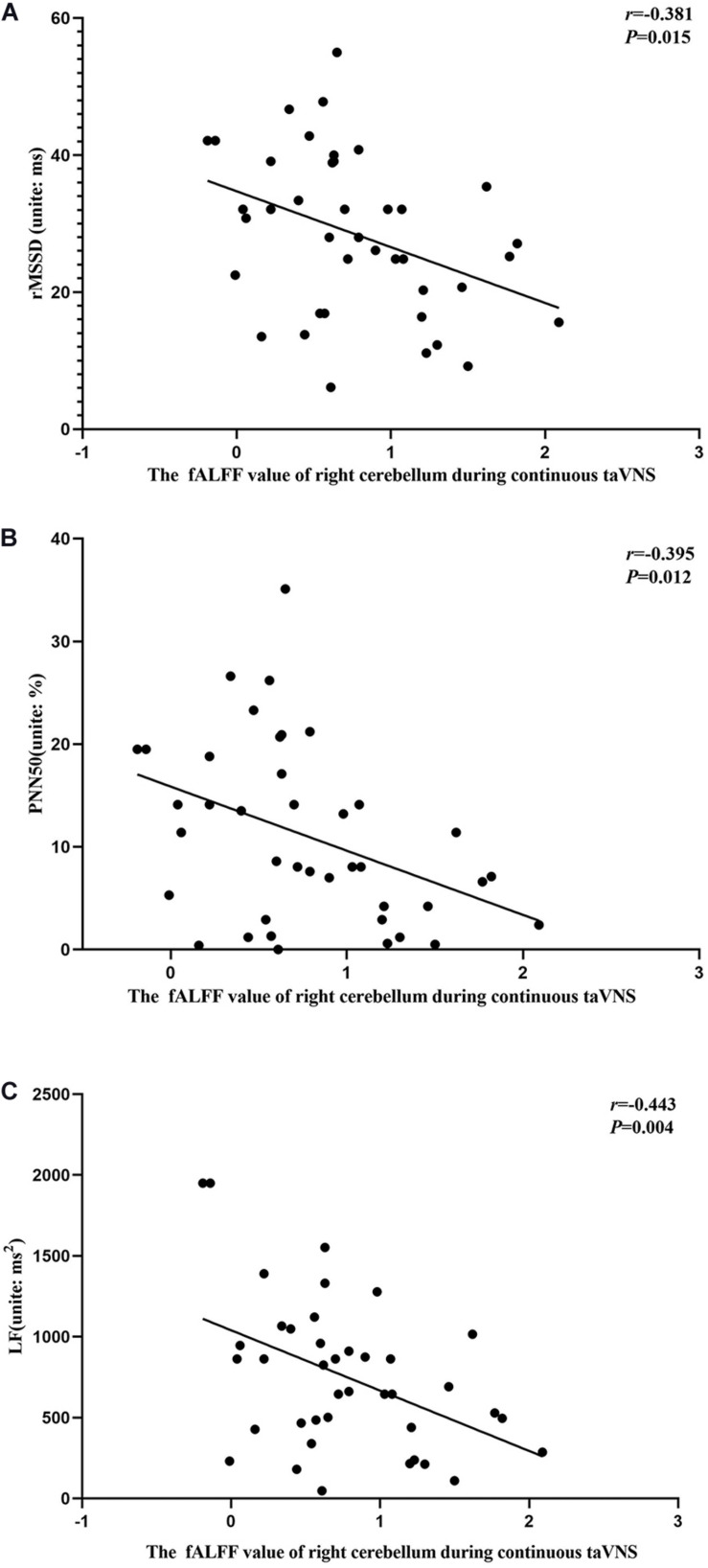
Results of the correlation analysis between fractional amplitude of low-frequency fluctuation (fALFF) values of the right cerebellum and heart rate variability parameters during continuous transcutaneous auricular vagus nerve stimulation state. **(A–C)** Correlations between the fALFF value of the right cerebellum and rMSSD **(A)**, PNN50 **(B)**, and LF **(C)**.

**FIGURE 9:**
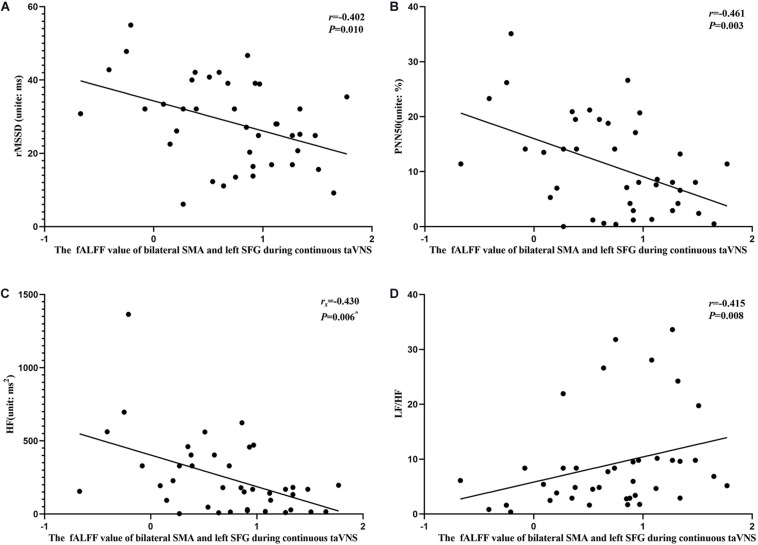
Results of the correlation analysis between heart rate variability parameters and fractional amplitude of low-frequency fluctuation (fALFF) values of the bilateral supplementary motor area (SMA) and left superior frontal gyrus (SFG) during continuous transcutaneous auricular vagus nerve stimulation state. **(A–D)** Correlations between the fALFF value of the bilateral SMA and left SFG and rMSSD **(A)**, PNN50 **(B)**, HF **(C)**, and LF/HF **(D)**. The asterisk means *P*-value for Spearman’s correlation analysis.

The results of the correlation analyses showed that the clinical efficacy of taVNS efficacy after 4 weeks of treatment and the HRV and fALFF values during continuous taVNS state were interrelated (Figures 6–9). Specifically, the efficacy (difference in PSQI score before *vs* after 1 month of taVNS) was correlated with the fALFF value of the right cerebellum (*r* = –0.574, *P* < 0.001) and the combination of bilateral SMA and left SFG (*r* = –0.647, *P* < 0.001). On the value of HRV indicators, the efficacy was correlated with rMSSD (*r* = 0.385, *P* = 0.014), PNN50 (*r* = 0.413, *P* = 0.008), LF (*r* = 0.314, *P* = 0.048), and HF (*r*s = 0.411, *P* = 0.008). For the association between HRV and fALFF value, the fALFF value of the right cerebellum was correlated with rMSSD (rs = –0.381, *P* = 0.015), PNN50 (*r* = –0.395, *P* = 0.012), and LF (*r* = –0.443, *P* = 0.004); the fALFF value of the bilateral SMA and left SFG was correlated with rMSSD (*r* = –0.402, *P* = 0.010), PNN50 (*r* = –0.461, *P* = 0.003), HF (*r*s = –0.430, *P* = 0.006), and LF/HF (*r* = –0.415, *P* = 0.008).

## Discussion

In this study, we found that, during continuous taVNS state, group A had a lower brain activity in the SMN and an elevated autonomic activity than group B as reflected by lower fALFF values and higher HRV parameter (HF, PNN50, and RMSSD) values, respectively. Moreover, we found that the efficacy indicators (PSQI score reduction rate and difference in PSQI score before *vs.* after treatment), HRV parameters, and fALFF values of the SMN (right cerebellum, right mSFG, and bilateral SMA) during continuous taVNS state were interrelated, suggesting that the fALFF values of the SMN and the HRV value can serve as biomarkers for the efficacy of taVNS treatment for PI.

The efficacy of taVNS in the treatment of insomnia has been previously investigated. taVNS is thought to promote the secretion of melatonin or γ-aminobutyric acid, which has a sedative effect (Luo et al., 2017). Vagus nerve stimulation decreases the excitability of the hypothalamic–pituitary–adrenal axis (O’Keane et al., 2005), regulates heart rate and basal metabolic rate (Val-Laillet et al., 2015; Kawada et al., 2019), alleviates negative mood, and facilitates the body’s transition to a sleep state. Sleep behaviors are regulated by cholinergic neurons of the vagus nerve (Mu and Huang, 2019); taVNS may improve sleep quality by directly regulating the activity of this nerve (Hallböök et al., 2005). Patients with insomnia often exhibit hyperactivation of the default mode network (DMN) and visual areas of the brain; taVNS was shown to inhibit activity in these brain regions, thereby alleviating sleep disorder and hypersensitivity to light (Zhao et al., 2020). Additionally, taVNS may relieve depression and anxiety symptoms that often accompany PI by regulating the activity of the amygdala and DMN (Rong et al., 2016). These findings provide insight into the mechanistic basis for the therapeutic effects of taVNS in PI.

In our study, taVNS alleviated insomnia in 70.13% of patients. Different efficacy rates for taVNS have been reported by different studies (Lan et al., 2015), with the quality of evidence from most of these studies found to be moderate (Tan et al., 2014). One study reported an efficacy rate of 80% for taVNS in PI patients (Li et al., 2019). The efficacy of taVNS also varies in other diseases for which this technique has been approved as a treatment by the FDA; for example, taVNS was effective in 66% of patients with drug-resistant epilepsy (Roldán et al., 2013), while in another study only 54% of patients with drug-resistant epilepsy experienced a decrease in seizure frequency and symptom relief after 24 weeks (Rong et al., 2014).

The observed inter-individual differences in the efficacy of taVNS may be due to the differential sensitivity of the vagus nerve to external stimulation. HRV is an objective and commonly used indicator of the autonomic nervous response (Chapleau and Sabharwal, 2011; Badran et al., 2018b; Williams et al., 2019) that can vary across individuals subjected to the same stimulation parameters (Ojeda et al., 2016). Changes in the activation of the vagus nerve are reflected as alterations in the values of HRV parameters (HF, RMSSD, and PNN50) (Yamanaka et al., 2015). Patients with drug-resistant epilepsy with a higher HRV value during VNS showed a better response than those with a lower value (Liu et al., 2018). Thus, HRV is associated with the efficacy of taVNS. We also found that the values of HRV parameters (HF, PNN50, and RMSSD) were higher for group A than for group B during continuous taVNS state despite there being no difference between the two groups before the stimulation. These results suggest that taVNS is more effective in patients whose autonomic nervous system is more sensitive to stimulation and that the efficacy of taVNS can be evaluated based on HRV parameters.

taVNS targets the brain *via* the auricular vagus nerve, which is part of the afferent vagus nerve (Yakunina et al., 2017; Hachem et al., 2018; Badran et al., 2019). The auricular vagus nerve regulates the activity of the autonomic nervous system, which shows differences in sensitivity to VNS, depending on the patient (Javaheri and Redline, 2017). The vagus nerve is a mixed fiber containing both sensory and motor neurons (Butt et al., 2020); its stimulation alters the activity of sensory and motor brain regions that are closely related to the SMN (Edgerton and Gad, 2018). taVNS has been shown to modulate the activity of the SMN; for example, taVNS attenuated the hyperactivation of the SMN in insomnia patients (Zhao et al., 2020). Moreover, taVNS caused greater changes in the activity of SMN regions than stimulation of non-vagus nerves (Badran et al., 2018a). As the SMN is associated with the occurrence of insomnia and the sensitivity of the vagus nerve, it is the likely brain region that is influenced by taVNS. We speculated that the differential regulation of the SMN and autonomic nervous system constitutes the mechanistic basis for the variable efficacy of taVNS in PI. This was confirmed by the observed correlations between treatment efficacy and HRV and fALFF values of some brain regions in the SMN during continuous taVNS state. Additionally, the fALFF values of the SMN were lower for group A (effective group) than for group B (ineffective group). Thus, the fALFF values of the SMN along with HRV value during continuous taVNS state may reflect the efficacy of taVNS for insomnia treatment. PI patients often have increased ALFF or fALFF values of the SMN; abnormal SMN activity often manifests as excessive sensitivity to sound, light, and physical stimulation (Dai et al., 2016). CBT-I was shown to decrease the ALFF value of the SMN in insomnia patients (Kim et al., 2017). In clinical practice, doctors could screen patients who are sensitive to taVNS before initiating the treatment in order to increase the treatment success rate and avoid wasting medical resources.

Our studies had some limitations. Firstly, in our 77 insomnia patients, only 20 patients had a score reduction rate of PSQI < 25%, which had relatively poor efficacy. In order to make the differences of patients belonging to a different efficacy more significant and obvious, we only use the top 20 patients in efficacy to compare with the patients with relatively poor efficacy, so our sample size was relatively small, and we did not determine the threshold values of indicators that are closely related to efficacy and could predict the efficacy. Secondly, we used standard stimulation parameters employed by other investigators and did not examine the influence of different parameters on patients who are sensitive to taVNS stimulation. These points will be addressed in future studies.

## Conclusion

The results of our study demonstrate that patients with PI have variable sensitivity to continuous taVNS, which can be attributed to differences in the regulation of the SMN and autonomic nervous system responses. Given their correlations with PSQI score reduction rate, HRV and fALFF are potential biomarkers for predicting the clinical outcome of PI treated by taVNS.

## Data Availability Statement

The raw data supporting the conclusions of this article will be made available by the authors, without undue reservation.

## Ethics Statement

The studies involving human participants were reviewed and approved by Ethics Committee of Guangdong Provincial Hospital of Chinese Medicine. The patients/participants provided their written informed consent to participate in this study.

## Author Contributions

BL, XW, and B-yX designed the study. XW, B-yX, R-rM, and W-tL acquired the data. YZ, XW, BL, W-tL, and X-yH performed the data analysis. XW, W-tL, YZ, and R-rM interpreted the results. XW, BL, YZ, and W-tL prepared the manuscript. All the authors contributed to manuscript revision and approved the final version for publication.

## Conflict of Interest

The authors declare that the research was conducted in the absence of any commercial or financial relationships that could be construed as a potential conflict of interest.
